# Risk of Vertebral Fracture in Patients Diagnosed with a Depressive Disorder: A Nationwide Population-Based Cohort Study

**DOI:** 10.6061/clinics/2017(01)08

**Published:** 2017-01

**Authors:** Shyh-Chyang Lee, Li-Yu Hu, Min-Wei Huang, Cheng-Che Shen, Wei-Lun Huang, Ti Lu, Chiao-Lin Hsu, Chih-Chuan Pan

**Affiliations:** I Kaohsiung Veterans General Hospital, Department of Psychiatry, Kaohsiung, Taiwan.; II Taichung Veterans General Hospital, Department of Orthopedics, Chiayi Branch, Chiayi, Taiwan.; III National Yang-Ming University, Faculty of Medicine, Division of Psychiatry, Taipei, Taiwan.; IV Taichung Veterans General Hospital, Department of Psychiatry, Chiayi Branch, Chiayi, Taiwan.; V National Chung-Cheng University, Department of Information Management, Chiayi, Taiwan.; VI Taipei Veteran General Hospital, Department of Family Medicine, Taitung Branch, Taitung, Taiwan; VII Kaohsiung Veterans General Hospital, Department of Family Medicine, Kaohsiung, Taiwan.; VIII Kaohsiung Veterans General Hospital, Physical Examination Center, Kaohsiung, Taiwan.

**Keywords:** Depressive Disorder, Osteoporotic Fracture, Vertebral Fracture, Risk Factor

## Abstract

**OBJECTIVE:**

Previous studies have reported that depression may play a crucial role in the occurrence of vertebral fractures. However, a clear correlation between depressive disorders and osteoporotic fractures has not been established. We explored the association between depressive disorders and subsequent new-onset vertebral fractures. Additionally, we aimed to identify the potential risk factors for vertebral fracture in patients with a depressive disorder.

**METHODS:**

We studied patients listed in the Taiwan National Health Insurance Research Database who were diagnosed with a depressive disorder by a psychiatrist. The comparison cohort consisted of age- and sex-matched patients without a depressive disorder. The incidence rate and hazard ratios of subsequent vertebral fracture were evaluated. We used Cox regression analysis to evaluate the risk of vertebral fracture among patients with a depressive disorder.

**RESULTS:**

The total number of patients with and without a depressive disorder was 44,812. The incidence risk ratio (IRR) between these 2 cohorts indicated that depressive disorder patients had a higher risk of developing a subsequent vertebral fracture (IRR=1.41, 95% confidence interval [CI]=1.26–1.57, *p*<0.001). In the multivariate analysis, the depressive disorder cohort showed a higher risk of vertebral fracture than the comparison cohort (adjusted hazard ratio=1.24, 95% CI=1.11–1.38, *p*<0.001). Being older than 50 years, having a lower monthly income, and having hypertension, diabetes mellitus, cerebrovascular disease, chronic obstructive pulmonary disease, autoimmune disease, or osteoporosis were considered predictive factors for vertebral fracture in patients with depressive disorders.

**CONCLUSIONS:**

Depressive disorders may increase the risk of a subsequent new-onset vertebral fracture.

## INTRODUCTION

Vertebral fractures are a critical public health problem and can result in acute and chronic pain, decreased quality of life, and diminished lifespans 1. One in six women and one in twelve men will sustain a symptomatic vertebral fracture in their lifetime 2. In Taiwan, the prevalence of vertebral fracture in women 65 years and older has been reported to be 19.8%, which was higher than the 12.5% in men of the same age group 3. Many vertebral fracture patients do not receive medical attention; only a fourth to a third of vertebral fractures are clinically diagnosed 4. The risk factors for vertebral fracture include advanced age, female sex, dementia, susceptibility to falling, a history of fracture, alcohol consumption, tobacco use, osteoporosis, estrogen deficiency, impaired eyesight, insufficient physical activity, low body weight, dietary calcium deficiency, and vitamin D deficiency 5,6.

Depressive disorders are common psychiatric problems in modern society and are some of the most substantial causes of disease burden in the general population 7. Depressive disorders are clinically heterogeneous disorders that are thought to result from an interaction of multiple genes with environmental influences as well as with developmental epigenetic components 8. Hyperintense lesions in brain magnetic resonance imaging scans have been associated with depressive disorders, especially in late life depression, and have led to the development of the theory of vascular depression 9,10. Meta-analytic studies have provided strong evidence that depressed patients have lower levels of brain-derived neurotrophic factor (BDNF) than healthy controls and have found significantly higher BDNF levels after antidepressant treatment 11,12. However, the exact underlying pathophysiological mechanisms of depressive disorders remain unclear.

Previous studies have demonstrated that depressive symptoms and likely depressive disorders are frequently observed in patients suffering from vertebral fractures 13-15. The extent of preoperative depression is an independent predictor of less functional improvement following revision lumbar surgery 16. The long-term complications of vertebral fractures include kyphosis, deconditioning, insomnia, and depression 17. However, studies on the relationship between depressive disorders and the incidence of vertebral fracture have yielded conflicting results 18,19.

There is a lack of national data on this subject, and few longitudinal studies have been conducted on the association between depressive disorders and the subsequent risk of vertebral fracture. Therefore, the aim of our study was to determine whether depressive disorders increase the incidence of vertebral fracture. This nationwide population-based retrospective cohort study involved data derived from the National Health Insurance (NHI) system in Taiwan. The independent risk factors of vertebral fracture in patients with depressive disorders were also investigated.

## MATERIALS AND METHODS

### Data Source

The National Health Insurance Research Database (NHIRD) was established in 1996 in Taiwan, and by the end of 2007, this database contained the registry files and all medical benefit claims for nearly 99% of the residents in Taiwan. Numerous researchers worldwide have used the NHIRD. The NHIRD contains substantial information regarding clinic visits, including the date, prescription details, and diagnostic codes using the International Classification of Diseases, Ninth Revision, Clinical Modification (ICD-9-CM). The NHIRD was developed and is managed by the National Health Research Institutes (NHRI), with the Bureau of National Health Insurance maintaining the confidentiality of the data.

The Longitudinal Health Insurance Database 2000 (LHID 2000) was used in this study and comprises a systematic and random sampling of the NHIRD. The LHID 2000 contains all of the original claims data for one million beneficiaries who were randomly sampled from the 2000 Registry of Beneficiaries of the NHIRD, which included the registration data of all beneficiaries of the NHI program from January 1, 2000, to January 1, 2001. Approximately 25.68 million individuals are in this registry. We obtained data from 1996-2013 for the one million individuals in the LHID 2000. The LHID 2000 also contains historical ambulatory data for the one million randomly sampled beneficiaries enrolled in the NHIRD between 1996 and 2000. The LHID 2000 updates the medical records of the enrolled patients each year; therefore, it contains medical information on patients from 2000 to 2013. The NHRI confirmed that there were no significant differences in the distributions of age, sex, or health care costs between the beneficiaries in the LHID and those in the NHIRD.

### Ethics Statement

This study was approved by the Institutional Review Board (IRB) of the Kaohsiung Veterans General Hospital (No: VGHKS14-CT7-07). Written consent was not obtained from the study participants, because the data were obtained from the LHID 2000, which contains deidentified data. In addition, the Institutional Review Board issued a formal written waiver of the need for consent.

### Study Population

We extracted data from the LHID 2000 for this retrospective cohort study, which investigated patients newly diagnosed with vertebral fracture between January 1, 2000, and December 31, 2013. Patients diagnosed with a depressive disorder between January 1, 2000, and December 31, 2004, were defined according to the ICD-9-CM codes 296.2–296.3, 300.4, and 311. To ensure diagnostic validity and patient homogeneity, we included only patients who were diagnosed by psychiatrists. We excluded patients who were diagnosed with a vertebral fracture between January 1, 1996, and December 31, 1999. For each patient with a depressive disorder included in the final cohort, 4 age- and sex-matched patients without a depressive disorder were randomly selected from the LHID 2000. The index date was defined as the first date in the database that indicated a depressive disorder diagnosis, and we began following both patients with a depressive disorder and the matched cohort from that time. All participants were observed until they were diagnosed with vertebral fracture (ICD-9-CM codes: 805.2, 805.4, 805.6, 805.8, 806.2, and 806.4) or until death, withdrawal from the NHI system, or December 31, 2013. The primary clinical outcome was vertebral fracture. Furthermore, common comorbidities, including hypertension, coronary artery disease, diabetes mellitus, dyslipidemia, cerebrovascular disease, chronic obstructive pulmonary disease (COPD), nephropathy, chronic liver disease, autoimmune disease, and osteoporosis, were compared between participants in the depressive disorder and in the control cohorts.

### Statistical Analysis

The incidence of newly diagnosed vertebral fracture in the depressive disorder and control cases was the primary outcome in this study. We compared the distributions of the demographic characteristics between the 2 groups using independent *t* tests and chi-square tests. To investigate potential surveillance bias, subgroups were stratified according to the duration since the diagnosis of a depressive disorder. Furthermore, a Cox proportional hazard regression model was used to calculate the hazard ratios (HRs) of vertebral fracture in the depressive disorder and control cohorts.

SAS software for Windows, Version 9.3 (SAS Institute, Cary, NC, USA), was used for the data extraction, computation, linkage, processing, and sampling. All other statistical analyses were performed using SPSS software for Windows, Version 20 (IBM, Armonk, NY, USA); *p*<0.05 was considered statistically significant.

## RESULTS

Figure 1 displays the flowchart of enrollment. Our study sample comprised 11,203 patients with a depressive disorder and 44,812 control patients. The basic characteristics of the 2 cohorts are shown in Table 1. The median age was 38.6 years (interquartile range, 28.6–50.9 years). Compared with the control cohort, the depressive disorder cohort had higher percentages of comorbidities, a significantly lower income, and a lower degree of urbanization.

As shown in Table 2, during the entire follow-up period, 459 patients with a depressive disorder and 1335 control patients were diagnosed with a vertebral fracture. The incidence risk ratio (IRR) of vertebral fracture was significantly higher in patients with a depressive disorder than in the controls (IRR=1.41, 95% CI=1.26–1.57, *p*<0.001). Stratification according to age and sex revealed that the IRR of new-onset vertebral fracture remained significantly higher in both the female and male groups, as well as in different age groups. In patients with a depressive disorder, fractures occurred most frequently during the period 5–10 years after diagnosis, followed by 1–5 years after in both groups. Vertebral fracture events decreased after the follow-up period had exceeded 10 years for both groups. Furthermore, stratification by follow-up duration revealed a significantly higher IRR of vertebral fracture in the 5- to 10-year follow-up period only (IRR=1.26, 95% CI=1.06–1.48, *p*=0.006).

Table 3 presents a comparison of the crude HRs and adjusted hazard ratios (aHRs) of the newly diagnosed vertebral fracture for patients with a depressive disorder and for controls. The results of the multivariate analysis indicated that patients with a depressive disorder had a significantly higher risk of a subsequent vertebral fracture (aHR=1.24, 95% CI=1.11–1.38, *p*<0.001).

In addition, multivariate analyses were performed using a Cox regression model to identify the potential risk factors for vertebral fracture in patients with a depressive disorder; as shown in Table 4, the results of these analyses showed that the predictive factors for vertebral fracture in patients suffering from a depressive disorder included being older than 50 years old, having a lower monthly income, and having hypertension, diabetes mellitus, cerebrovascular disease, COPD, autoimmune disease, or osteoporosis.

## DISCUSSION

The current study indicated that based on a nationwide population-based dataset, depressive disorders were associated with an increased sequential risk of vertebral fracture. In addition, the risk factors for developing subsequent vertebral fracture in patients with a depressive disorder included being 50 years old or older, being female, and having hypertension, diabetes mellitus, cerebrovascular disease, COPD, autoimmune disease, or osteoporosis.

Previous studies have demonstrated a positive relationship between depressive disorders and a higher risk of vertebral fracture 18,20,21, but other research has reported different findings 19,22. However, our results support the notion that the risk of vertebral fracture is significantly higher in patients with a depressive disorder. Our study was improved by conducting a nationwide longitudinal analysis with a large sample size and a long follow-up duration. Furthermore, participation in the NHI is mandatory and involves an unbiased participant selection process; moreover, all residents in Taiwan can obtain low-cost health care. Finally, the referral bias was low and the follow-up compliance high in the cohort studied.

We found in this study that most incident fractures occurred during the 5–10 year follow-up period and decreased thereafter. This may be due to the fact that women’s lumbar spine bone mineral density (BMD) values are significantly lower after menopause and subsequently decrease even further. Another possibility is that the participants died after 10 years of follow-up 23.

Several possible mechanisms could underlie the increased risk of vertebral fracture observed among patients with a depressive disorder. First, the development of a vertebral fracture after acquiring a depressive disorder might be caused by a lower BMD in depressive disorder patients. Some research has demonstrated that major depression and depressive symptoms adversely affect bone density and result in an increased fracture risk 24-26. In addition, previous studies have suggested that depressive disorders result in dysregulation of the hypothalamic-pituitary-adrenocortical axis, the sympathoadrenal axis, parathyroid hormones, and cytokines, and these disruptions may provide insight into the relationship between osteoporosis and depression 25,27,28. Other possible pathophysiological factors underlying osteopenia in depressive disorder patients include their immune response, use of alcohol, poor nutritional status, social circumstances, and unhealthy lifestyles 29-31. These factors are consistent with the results of our study. Osteoporosis is a significant risk factor for vertebral fracture in patients with a depressive disorder. Second, neuropathological lesions in certain regions of the brain in patients with depressive disorders may increase the risk of falls and result in vertebral fracture. Previous findings support the hypothesis that mood disorders involve dysfunction of the limbic system, the basal ganglia, and the hypothalamus. Other neuroscience studies have focused on brain regions including the prefrontal cortex, the anterior cingulate, the hippocampus, and the amygdala 32. Some studies have shown that patients with depressive disorders experience a reduction in the gray matter volume of specific brain regions 33, morphological atrophy caused by excess neuron loss 34, vascular atherosclerosis, and neurodegenerative damage 35. These pathophysiological impairments of the brain can influence these patients’ balance, judgment, gait, and coordination. Third, psychotropic medications (e.g., antidepressants and/or benzodiazepines), which are widely used in depressed patients, may play a critical role in the risk of vertebral fracture, especially in the geriatric population. This potential association is consistent with our finding that age (aged 50 years and older) was strongly linked to an increase in vertebral fracture risk in patients with a depressive disorder. Bolton et al. reported that selective serotonin reuptake inhibitors (SSRIs) and benzodiazepines were associated with greater risks of falls and fractures in a dose–effect relationship 36. Antidepressants have been associated with increased bone loss and fracture through the activation of 5-HT receptors on osteoblasts and osteoclasts via endocrine, autocrine/paracrine and neuronal pathways 37,38. Other studies have suggested that all antidepressant classes (tricyclic antidepressants, SSRIs, and other antidepressants) are associated with greater risks of falls and fractures 39-41. Another finding in our study was that the risk of vertebral fracture was significantly higher in low-income populations. Previous studies have reported that rural populations have a higher level of poverty, lower values of vitamin D, lower BMD in the lumbar spine, a higher prevalence of vertebral fracture, and a higher prevalence of osteoporosis than urban populations 42,43. Many studies have provided evidence that having a low income is associated with an increased risk of lower BMD at all skeletal sites and have found higher fracture rates in lower income groups 44-46. Research has highlighted the need to urgently improve the nutritional status, dietary calcium intake, bone health, and living environment of low-income populations and to provide them with adequate opportunities for osteoporosis treatment 47-49. In our study, compared with the age- and sex-matched controls, the risk of vertebral fracture was higher among low-income patients with a depressive disorder. Additionally, our study demonstrated that there was no difference in the risk of vertebral fracture between patients with a depressive disorder according to urbanization, as shown in Table 4. Previous research on patients with a traumatic fracture has claimed that the activities associated with a rural lifestyle result in more injuries and that an urban lifestyle results in a lower fracture risk 50. Another study on postmenopausal women living in rural and urban areas revealed a different perspective from that of our study, stating that rural populations experience more poverty and have lower values of vitamin D, lower BMD in the lumbar spine, and a higher prevalence of vertebral fracture and osteoporosis 42. In addition, one study evaluated femoral neck BMD in rural residents and found that the incidence of osteoporosis was higher in rural participants than in their urban counterparts, thus explaining the urban-rural difference in fracture incidence 51. We suspect that this finding may be due to the fact that rural participants seldom seek medical advice, resulting in a decreased vertebral fracture incidence rate.

Our study is one of the few nationwide cohort studies to evaluate the association between depressive disorders and subsequent vertebral fracture. However, it has several limitations inherent to the use of claims databases that should be considered. First, pharmacy data were not analyzed in our study, and these data may provide a crucial connection between depressive disorders and the risk of vertebral fracture. Second, several demographic variables were not available, such as alcohol consumption, tobacco use, lifestyle, family history of a depressive disorder or of vertebral fracture, and body mass index. These factors may influence the risk of vertebral fracture in patients with a depressive disorder. Third, we used ICD-9 codes to define depressive disorders and vertebral fracture. The prevalence rate may have been underestimated because only patients seeking medical services could be identified through the database. These identification problems might have led to an underestimation of the association between depressive disorders and vertebral fractures. Fourth, the data from the NHIRD were stored for health services billing purposes and did not undergo verification for scientific research.

In conclusion, the results of this study suggest that depressive disorders increase the risk of vertebral fractures. The relationship remained significant after adjusting for selected confounding factors including age, sex, and underlying medical comorbidities. Furthermore, in depressed patients, the risk of vertebral fracture was higher in older patients (50 years and above), female patients, those with hypertension, diabetes mellitus, cerebrovascular disease, COPD, autoimmune disease, and osteoporosis, and those with a low income. Adequate prevention strategies are essential to reducing the incidence of vertebral fracture in this high-risk group of depressive disorder patients. Additional prospective clinical cohort studies on the relationship between depressive disorders and subsequent vertebral fracture are needed.

## AUTHOR CONTRIBUTIONS

Pan CC and Lee SC were the leaders of the project. They contributed to the organization of all the study processes, supervised all co-authors´ work and provided instructions during the study. Huang MW and Hu LY participated in the design of the study and analyzed the data. Shen CC, Huang WL and Lu T contributed to the interpretation of the data and confirmed the accuracy of the data. Hsu CL primarily helped to write the original manuscript. All co-authors read and approved the submitted manuscript.

## Figures and Tables

**Table 1:** Baseline Characteristics of Patients with and without Depression.

Demographic data	Patients with Depression *n*=11,203		Patients without Depression *n*=44,812		*p* value
	*N*	%	*n*	%	
Age (years)^a^	38.6 (28.6–50.9)		38.6 (28.6–50.9)		
≥50	2,968	26.5	11,872	26.5	0.999
<50	8,235	73.5	32,940	73.5	
Sex, No. (%)					
Male	4,501	40.2	18,004	40.2	0.999
Female	6,702	59.8	26,808	59.8	
Comorbidities, No. (%)					
Hypertension	2,504	22.4	7,229	16.1	<0.001
Coronary artery disease	1,705	15.2	4,089	9.1	<0.001
Diabetes mellitus	1,596	14.2	4,426	9.9	<0.001
Dyslipidemia	2,071	18.5	5,637	12.6	<0.001
Cerebrovascular disease	1,291	11.5	2,757	6.2	<0.001
COPD	1,461	13.0	3,645	8.1	<0.001
Nephropathy	1,437	12.8	3,437	7.7	<0.001
Chronic liver disease	3,844	34.3	9,561	21.3	<0.001
Autoimmune disease	701	6.3	1,874	4.2	<0.001
Osteoporosis	735	6.6	1,793	4.0	<0.001
Urbanization, No. (%)					<0.001
Urban	7,150	63.8	28,142	62.8	
Suburban	3,173	28.3	13,905	31.0	
Rural	880	7.9	2,765	6.2	
Monthly Income, No. (%)					<0.001
>NT$42,000^b^	1,145	10.2	4,602	10.3	
NT$19,100–NT$42,000^b^	1,968	17.6	9,106	20.3	
NT$0–NT$19,000^b^	5,617	50.1	21,489	48.0	
Dependent	2,473	22.1	9,615	21.5	
Follow-up years^a^	10.77 (9.51–12.23)		10.86 (9.64–12.28)		<0.001

a Median age (interquartile range)

b NT$1= US$0.033.

COPD, chronic obstructive pulmonary disease.

**Table 2:** Incidence of Vertebral Fracture in Patients with and without Depression.

	Patients with Depression		Patients without Depression		Risk ratio (95% CI)	*p* value
	No. of Vertebral Fracture	Per 1,000 person-years	No. of Vertebral Fracture	Per 1,000 person-years		
Total	459	3.97	1,335	2.82	1.41 (1.26–1.57)	<0.001
Age						
≥50	274	10.17	898	7.87	1.29 (1.12–1.48)	<0.001
<50	185	2.09	437	1.22	1.72 (1.44–2.04)	<0.001
Sex						
Male	138	3.07	388	2.08	1.48 (1.21–1.80)	<0.001
Female	321	4.54	947	3.30	1.38 (1.21–1.56)	<0.001
Follow-up, y						
0–1	42	400.00	112	362.46	1.10 (0.75–1.59)	0.580
1–5	171	89.30	426	79.40	1.12 (0.94–1.35)	0.197
5–10	194	7.18	615	5.71	1.26 (1.06–1.48)	0.006
≥10	52	0.60	182	0.51	1.19 (0.86–1.63)	0.274

CI, confidence interval.

**Table 3:** Analyses of Risk Factors for Vertebral Fracture in Patients with and without Depression.

Predictive variables	Univariate analysis		Multivariate analysis	
	HR (95% CI)	*p* value	HR (95% CI)	*p* value
Depression	1.41 (1.27–1.57)	<0.001	1.24 (1.11–1.38)	<0.001
Age (≥50 = 1, <50 = 0)	6.05 (5.49–6.67)	<0.001	4.12 (3.67–4.63)	<0.001
Sex (Female = 1, Male = 0)	1.56 (1.41–1.72)	<0.001	1.62 (1.46–1.80)	<0.001
Comorbidities				
Hypertension	3.86 (3.52–4.25)	<0.001	1.33 (1.18–1.50)	<0.001
Coronary artery disease	3.37 (3.03–3.75)	<0.001	1.15 (1.02–1.30)	0.028
Diabetes mellitus	3.07 (2.76–3.42)	<0.001	1.20 (1.05–1.36)	0.006
Dyslipidemia	2.72 (2.46–3.01)	<0.001	0.97 (0.86–1.10)	0.678
Cerebrovascular disease	3.37 (2.99–3.80)	<0.001	1.29 (1.13–1.47)	<0.001
COPD	2.33 (2.06–2.63)	<0.001	1.08 (0.94–1.23)	0.271
Nephropathy	2.48 (2.20–2.81)	<0.001	1.27 (1.11–1.45)	<0.001
Chronic liver disease	1.83 (1.66–2.01)	<0.001	1.14 (1.03–1.27)	0.016
Autoimmune disease	2.08 (1.76–2.45)	<0.001	1.38 (1.16–1.63)	<0.001
Osteoporosis	4.17 (3.67–4.75)	<0.001	1.35 (1.17–1.55)	<0.001
Degree of urbanization				
Urban	Reference			
Suburban	1.24 (1.12–1.37)	<0.001	1.12 (1.01–1.25)	0.027
Rural	1.98 (1.70–2.31)	<0.001	1.32 (1.13–1.55)	<0.001
Monthly Income				
>NT$42,000^a^	Reference		Reference	
NT$19,100–NT$42,000^a^	2.66 (2.15–3.30)	<0.001	1.58 (1.27–1.97)	<0.001
NT$0–NT$19,000^a^	2.25 (1.83–2.76)	<0.001	1.64 (1.33–2.02)	<0.001
Dependent	1.13 (0.89–1.43)	0.327	1.13 (0.89–1.43)	0.336

a NT$1= US$0.033.

HR, hazard ratio; CI, confidence interval; COPD, chronic obstructive pulmonary disease.

**Table 4:** Analyses of Risk Factors for Vertebral Fracture in Patients with Depression.

Predictive variables	Univariate analysis		Multivariate analysis	
	HR (95% CI)	*p* value	HR (95% CI)	*p* value
Age (≥50 = 1, <50 = 0)	4.91 (4.07–5.92)	<0.001	3.03 (2.38–3.84)	<0.001
Sex (Female = 1, Male = 0)	1.48 (1.21–1.80)	<0.001	1.56 (1.27–1.93)	<0.001
Comorbidities				
Hypertension	3.37 (2.80–4.05)	<0.001	1.38 (1.09–1.74)	0.008
Coronary artery disease	2.52 (2.06–3.09)	<0.001	0.92 (0.72–1.16)	0.474
Diabetes mellitus	3.03 (2.48–3.69)	<0.001	1.41 (1.11–1.79)	0.004
Dyslipidemia	2.49 (2.05–3.02)	<0.001	0.99 (0.78–1.26)	0.944
Cerebrovascular disease	3.26 (2.64–4.01)	<0.001	1.48 (1.17–1.86)	<0.001
COPD	2.37 (1.91–2.95)	<0.001	1.29 (1.03–1.63)	0.030
Nephropathy	1.87 (1.49–2.35)	<0.001	1.01 (0.79–1.29)	0.925
Chronic liver disease	1.66 (1.39–2.00)	<0.001	1.12 (0.92–1.37)	0.252
Autoimmune disease	2.10 (1.57–2.79)	<0.001	1.54 (1.15–2.06)	0.003
Osteoporosis	3.88 (3.09–4.88)	<0.001	1.44 (1.11–1.86)	0.005
Degree of urbanization				
Urban	Reference		Reference	
Suburban	1.12 (0.91–1.38)	0.288	1.02 (0.83–1.25)	0.870
Rural	1.76 (1.32–2.35)	<0.001	1.19 (0.89–1.61)	0.246
Monthly Income				
>NT$42,000^a^	Reference		Reference	
NT$19,100–NT$42,000^a^	2.02 (1.35–3.01)	<0.001	1.32 (0.88–1.98)	0.186
NT$0–NT$19,000^a^	1.82 (1.25–2.66)	0.002	1.53 (1.04–2.24)	0.031
Dependent	1.18 (0.76–1.84)	0.451	1.24 (0.80–1.93)	0.341

a NT$1= US$0.033.

HR, hazard ratio; CI, confidence interval; COPD, chronic obstructive pulmonary disease.
